# Sunlight

**Published:** 1891-08

**Authors:** 


### SUNLIGHT.

Houses in places otherwise unexceptionable are often so closely overhung with trees as to be in a state of humidity, owing to the prevention of a free circulation of air and a free admission of the sun's rays. Trees growing against the walls of houses, and shrubs in confined places near dwellings, are injurious also as favoring humidity. At the proper distance, on the other hand, trees are favorable to health. On this principle it may be understood how the inhabitants of one house suffer from various ills as the consequence of living in a confined, humid atmosphere, while their nearest neighbors, whose houses are otherwise situated, enjoy good health ; and even how one side of a large building fully exposed to the sun and a free circulation of air may be healthy, while the other side, overlooking shaded courts or gardens, is unhealthy.

